# 2-(4*H*-1,2,4-Triazol-4-yl)pyrimidine

**DOI:** 10.1107/S1600536811051968

**Published:** 2011-12-10

**Authors:** Qian Wang, Sheng Wang, Yuan Yuan Wang, Ying Wang

**Affiliations:** a Tianjin Key Laboratory of Structure and Performances for Functional Molecules, Tianjin Normal University, Tianjin 300387, People’s Republic of China

## Abstract

The title compound, C_6_H_5_N_5_, is almost planar, the triazole and pyrimidine rings forming a dihedral angle of 2.9 (13)°.

## Related literature

For the synthesis of the title compound, see: Wiley & Hart (1953 ▶). For properties of related compounds, see: Haasnoot (2000 ▶).
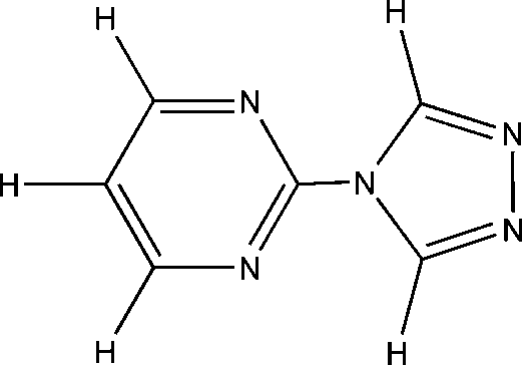


## Experimental

### 

#### Crystal data

C_6_H_5_N_5_
*M* *_r_* = 147.15
Triclinic, 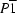

*a* = 5.6929 (10) Å
*b* = 7.7355 (14) Å
*c* = 8.6102 (15) Å
α = 67.233 (2)°
β = 80.755 (2)°
γ = 69.837 (2)°
*V* = 328.04 (10) Å^3^
*Z* = 2
Mo *K*α radiation
μ = 0.10 mm^−1^
*T* = 293 K
0.46 × 0.34 × 0.12 mm

#### Data collection

Bruker SMART CCD area-detector diffractometer
Absorption correction: multi-scan (*SADABS*; Sheldrick, 1996 ▶) *T*_min_ = 0.954, *T*_max_ = 0.988
1803 measured reflections
1154 independent reflections
916 reflections with *I* > 2σ(*I*)
*R*_int_ = 0.010

#### Refinement

*R*[*F* ^2^ > 2σ(*F* ^2^)] = 0.035
*wR*(*F* ^2^) = 0.101
*S* = 1.03
1154 reflections
100 parameters
H-atom parameters constrained
Δρ_max_ = 0.11 e Å^−3^
Δρ_min_ = −0.17 e Å^−3^

### 

Data collection: *SMART* (Bruker, 2008 ▶); cell refinement: *SAINT* (Bruker, 2008 ▶); data reduction: *SAINT*; program(s) used to solve structure: *SHELXS97* (Sheldrick, 2008 ▶); program(s) used to refine structure: *SHELXL97* (Sheldrick, 2008 ▶); molecular graphics: *ORTEP-3* (Farrugia, 1997 ▶); software used to prepare material for publication: *SHELXTL* (Sheldrick, 2008 ▶).

## Supplementary Material

Crystal structure: contains datablock(s) I, gllobal. DOI: 10.1107/S1600536811051968/aa2030sup1.cif

Structure factors: contains datablock(s) I. DOI: 10.1107/S1600536811051968/aa2030Isup2.hkl

Additional supplementary materials:  crystallographic information; 3D view; checkCIF report

## Figures and Tables

**Fig. 1.**
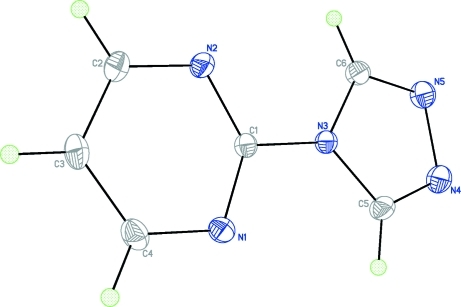
An ORTEP-3 view of the title compound with the displacement ellipsoids shown on 30% probability level.

## supplementary crystallographic information

### Comment

Many molecular compounds exhibit interesting magnetic and luminescent properties (Haasnoot, 2000). One of the requirements for posessing such macroscopic properties is to create interactions between the molecular units and the active sites within the crystal lattices. 1,2,4-Triazole and its derivatives are interesting bridging ligands. In the title compound the triazole and the pyrimidine rings are almost in the same plane, the dihedral angles between them is 2.9 (13)°.

### Experimental

A mixture of 1.2 g (0.012 mol) of pyrimidin-2-amine and 2.0 g (0.011 mol) of diformylhydrazine was heated slowly to 160–170 °C for 30 min. The crystals, which separated on cooling, were collected and recrystallized from water and acetonitrile and dried on air. Yield 0.7 g (43%). Anal. Calc. for C_6_H_5_N_5_ (%): C, 50.52; H, 5.30; N 44.18. Found (%): C, 50.59; H, 5.36; N 44.23.

### Refinement

Hydrogen atoms were included into calculated positions and refined as riding on the C atoms with C—H = 0.93 Å and *U*_iso_(H) = 1.2 or 1.5 *U*_eq_(C). Friedel pairs were averaged for the data used on the final cycles of the refinement.

### Crystal data

**Table:**

C_6_H_5_N_5_	*Z* = 2
*M**_r_* = 147.15	*F*(000) = 152
Triclinic, *P*1	*D*_x_ = 1.490 Mg m^−^^3^*D*_m_ = 1.490 Mg m^−^^3^*D*_m_ measured by not measured
Hall symbol: -P 1	Mo *K*α radiation, λ = 0.71073 Å
*a* = 5.6929 (10) Å	Cell parameters from 786 reflections
*b* = 7.7355 (14) Å	θ = 2.6–25.9°
*c* = 8.6102 (15) Å	µ = 0.10 mm^−^^1^
α = 67.233 (2)°	*T* = 293 K
β = 80.755 (2)°	Sheet, colourless
γ = 69.837 (2)°	0.46 × 0.34 × 0.12 mm
*V* = 328.04 (10) Å^3^	

### Data collection

**Table:**

Bruker SMART CCD area-detector diffractometer	1154 independent reflections
Radiation source: fine-focus sealed tube	916 reflections with *I* > 2σ(*I*)
graphite	*R*_int_ = 0.010
phi and ω scans	θ_max_ = 25.0°, θ_min_ = 2.6°
Absorption correction: multi-scan (*SADABS*; Sheldrick, 1996)	*h* = −6→6
*T*_min_ = 0.954, *T*_max_ = 0.988	*k* = −9→6
1803 measured reflections	*l* = −10→10

### Refinement

**Table:**

Refinement on *F*^2^	Primary atom site location: structure-invariant direct methods
Least-squares matrix: full	Secondary atom site location: difference Fourier map
*R*[*F*^2^ > 2σ(*F*^2^)] = 0.035	Hydrogen site location: inferred from neighbouring sites
*wR*(*F*^2^) = 0.101	H-atom parameters constrained
*S* = 1.03	*w* = 1/[σ^2^(*F*_o_^2^) + (0.0595*P*)^2^ + 0.0336*P*] where *P* = (*F*_o_^2^ + 2*F*_c_^2^)/3
1154 reflections	(Δ/σ)_max_ < 0.001
100 parameters	Δρ_max_ = 0.11 e Å^−^^3^
0 restraints	Δρ_min_ = −0.17 e Å^−^^3^

### Special details

**Table:**

Geometry. All s.u.'s (except the s.u. in the dihedral angle between two l.s. planes) are estimated using the full covariance matrix. The cell s.u.'s are taken into account individually in the estimation of s.u.'s in distances, angles and torsion angles; correlations between s.u.'s in cell parameters are only used when they are defined by crystal symmetry. An approximate (isotropic) treatment of cell s.u.'s is used for estimating s.u.'s involving l.s. planes.
Refinement. Refinement of *F*^2^ against ALL reflections. The weighted *R*-factor *wR* and goodness of fit *S* are based on *F*^2^, conventional *R*-factors *R* are based on *F*, with *F* set to zero for negative *F*^2^. The threshold expression of *F*^2^ > σ(*F*^2^) is used only for calculating *R*-factors(gt) *etc*. and is not relevant to the choice of reflections for refinement. *R*-factors based on *F*^2^ are statistically about twice as large as those based on *F*, and *R*- factors based on ALL data will be even larger.

### Fractional atomic coordinates and isotropic or equivalent isotropic displacement parameters (Å^2^)

**Table:**

	*x*	*y*	*z*	*U*_iso_*/*U*_eq_	
N1	0.5226 (2)	0.81660 (19)	0.85375 (15)	0.0447 (4)	
N2	0.2458 (2)	0.66148 (18)	0.82287 (15)	0.0450 (4)	
N3	0.20693 (19)	0.75348 (16)	1.05328 (14)	0.0371 (3)	
N4	0.0968 (3)	0.8138 (2)	1.28772 (16)	0.0564 (4)	
N5	−0.0695 (2)	0.7314 (2)	1.26282 (16)	0.0543 (4)	
C1	0.3340 (2)	0.74312 (19)	0.89977 (17)	0.0349 (3)	
C2	0.3638 (3)	0.6558 (2)	0.6765 (2)	0.0526 (4)	
H2	0.3095	0.6009	0.6157	0.063*	
C3	0.5620 (3)	0.7281 (2)	0.61289 (19)	0.0525 (4)	
H3	0.6422	0.7236	0.5109	0.063*	
C4	0.6357 (3)	0.8070 (2)	0.7069 (2)	0.0511 (4)	
H4	0.7702	0.8562	0.6671	0.061*	
C5	0.2575 (3)	0.8247 (2)	1.16196 (19)	0.0483 (4)	
H5	0.3898	0.8746	1.1478	0.058*	
C6	0.0005 (3)	0.6975 (2)	1.12413 (19)	0.0464 (4)	
H6	−0.0795	0.6422	1.0787	0.056*	

### Atomic displacement parameters (Å^2^)

**Table:**

	*U*^11^	*U*^22^	*U*^33^	*U*^12^	*U*^13^	*U*^23^
N1	0.0397 (6)	0.0550 (8)	0.0439 (7)	−0.0210 (6)	0.0051 (5)	−0.0192 (6)
N2	0.0507 (7)	0.0556 (8)	0.0394 (7)	−0.0242 (6)	0.0061 (5)	−0.0245 (6)
N3	0.0385 (6)	0.0415 (7)	0.0347 (7)	−0.0148 (5)	0.0037 (5)	−0.0172 (5)
N4	0.0665 (9)	0.0667 (9)	0.0457 (8)	−0.0241 (7)	0.0086 (6)	−0.0314 (7)
N5	0.0534 (8)	0.0674 (10)	0.0471 (8)	−0.0232 (7)	0.0136 (6)	−0.0278 (7)
C1	0.0347 (7)	0.0347 (8)	0.0336 (7)	−0.0089 (6)	0.0000 (5)	−0.0126 (6)
C2	0.0641 (10)	0.0598 (10)	0.0415 (9)	−0.0213 (8)	0.0045 (7)	−0.0266 (8)
C3	0.0561 (10)	0.0581 (10)	0.0371 (9)	−0.0134 (8)	0.0107 (7)	−0.0190 (8)
C4	0.0415 (8)	0.0609 (10)	0.0456 (9)	−0.0190 (8)	0.0097 (7)	−0.0151 (8)
C5	0.0556 (9)	0.0580 (10)	0.0448 (9)	−0.0256 (8)	0.0040 (7)	−0.0278 (8)
C6	0.0430 (8)	0.0593 (10)	0.0449 (9)	−0.0233 (7)	0.0090 (6)	−0.0245 (7)

### Geometric parameters (Å, °)

**Table:**

N1—C1	1.3199 (18)	N5—C6	1.2927 (19)
N1—C4	1.3407 (19)	C2—C3	1.373 (2)
N2—C1	1.3185 (18)	C2—H2	0.9300
N2—C2	1.3386 (18)	C3—C4	1.368 (2)
N3—C6	1.3624 (18)	C3—H3	0.9300
N3—C5	1.3629 (18)	C4—H4	0.9300
N3—C1	1.4184 (17)	C5—H5	0.9300
N4—C5	1.2965 (19)	C6—H6	0.9300
N4—N5	1.3915 (19)		
			
C1—N1—C4	114.10 (13)	C4—C3—C2	116.74 (14)
C1—N2—C2	114.64 (13)	C4—C3—H3	121.6
C6—N3—C5	103.94 (12)	C2—C3—H3	121.6
C6—N3—C1	127.43 (12)	N1—C4—C3	122.95 (14)
C5—N3—C1	128.61 (12)	N1—C4—H4	118.5
C5—N4—N5	106.86 (12)	C3—C4—H4	118.5
C6—N5—N4	107.05 (12)	N4—C5—N3	111.03 (13)
N2—C1—N1	129.15 (13)	N4—C5—H5	124.5
N2—C1—N3	115.06 (12)	N3—C5—H5	124.5
N1—C1—N3	115.79 (12)	N5—C6—N3	111.12 (13)
N2—C2—C3	122.41 (14)	N5—C6—H6	124.4
N2—C2—H2	118.8	N3—C6—H6	124.4
C3—C2—H2	118.8		
			
C5—N4—N5—C6	0.04 (17)	N2—C2—C3—C4	0.2 (2)
C2—N2—C1—N1	−1.0 (2)	C1—N1—C4—C3	0.0 (2)
C2—N2—C1—N3	178.77 (12)	C2—C3—C4—N1	−0.4 (2)
C4—N1—C1—N2	0.8 (2)	N5—N4—C5—N3	0.04 (18)
C4—N1—C1—N3	−179.02 (12)	C6—N3—C5—N4	−0.10 (17)
C6—N3—C1—N2	−3.3 (2)	C1—N3—C5—N4	178.59 (13)
C5—N3—C1—N2	178.33 (13)	N4—N5—C6—N3	−0.11 (17)
C6—N3—C1—N1	176.55 (13)	C5—N3—C6—N5	0.13 (17)
C5—N3—C1—N1	−1.8 (2)	C1—N3—C6—N5	−178.59 (13)
C1—N2—C2—C3	0.5 (2)		
